# Mechanisms of Magnetic Stimulation of Central Nervous System Neurons

**DOI:** 10.1371/journal.pcbi.1002022

**Published:** 2011-03-24

**Authors:** Tamar Pashut, Shuki Wolfus, Alex Friedman, Michal Lavidor, Izhar Bar-Gad, Yosef Yeshurun, Alon Korngreen

**Affiliations:** 1 The Leslie and Susan Gonda Multidisciplinary Brain Research Center, Bar-Ilan University, Ramat-Gan, Israel; 2 Department of Physics, Bar-Ilan University, Ramat-Gan, Israel; 3 Department of Psychology, Bar-Ilan University, Ramat-Gan, Israel; 4 The Mina and Everard Goodman Faculty of Life Sciences, Bar-Ilan University, Ramat-Gan, Israel; Université Paris Descartes, Centre National de la Recherche Scientifique, France

## Abstract

Transcranial magnetic stimulation (TMS) is a stimulation method in which a magnetic coil generates a magnetic field in an area of interest in the brain. This magnetic field induces an electric field that modulates neuronal activity. The spatial distribution of the induced electric field is determined by the geometry and location of the coil relative to the brain. Although TMS has been used for several decades, the biophysical basis underlying the stimulation of neurons in the central nervous system (CNS) is still unknown. To address this problem we developed a numerical scheme enabling us to combine realistic magnetic stimulation (MS) with compartmental modeling of neurons with arbitrary morphology. The induced electric field for each location in space was combined with standard compartmental modeling software to calculate the membrane current generated by the electromagnetic field for each segment of the neuron. In agreement with previous studies, the simulations suggested that peripheral axons were excited by the spatial gradients of the induced electric field. In both peripheral and central neurons, MS amplitude required for action potential generation was inversely proportional to the square of the diameter of the stimulated compartment. Due to the importance of the fiber's diameter, magnetic stimulation of CNS neurons depolarized the soma followed by initiation of an action potential in the initial segment of the axon. Passive dendrites affect this process primarily as current sinks, not sources. The simulations predict that neurons with low current threshold are more susceptible to magnetic stimulation. Moreover, they suggest that MS does not directly trigger dendritic regenerative mechanisms. These insights into the mechanism of MS may be relevant for the design of multi-intensity TMS protocols, may facilitate the construction of magnetic stimulators, and may aid the interpretation of results of TMS of the CNS.

## Author Summary

Transcranial magnetic stimulation (TMS) is a widely applied tool for probing cognitive function in humans and is one of the best tools for clinical treatments and interfering with cognitive tasks. Surprisingly, while TMS has been commercially available for decades, the cellular mechanisms underlying magnetic stimulation remain unclear. Here we investigate these mechanisms using compartmental modeling. We generated a numerical scheme allowing simulation of the physiological response to magnetic stimulation of neurons with arbitrary morphologies and active properties. Computational experiments using this scheme suggested that TMS affects neurons in the central nervous system (CNS) primarily by somatic stimulation. Since magnetic stimulation appears to cause somatic depolarization, its effects are highly correlated with the neuron's current threshold. Our simulations therefore predict that subpopulations of CNS neurons with different firing thresholds will respond differently to magnetic stimulation. For example, low-intensity TMS may be used to stimulate low-threshold cortical inhibitory interneurons. At higher intensities we predict that both inhibitory and excitatory neurons are activated. These predictions may be tested at the cellular level and may impact cognitive experiments in humans. Furthermore, our simulations may be used to design TMS coils, devices and protocols.

## Introduction

Noninvasive methods, such as electroencephalography (EEG), functional magnetic resonance imaging (fMRI) and magnetoencephalography (MEG), are commonly used to study the nervous system. Unlike these methods for passively recording neuronal activity, transcranial magnetic stimulation (TMS) actively stimulates neurons. A TMS coil is placed above the skull over a region of interest, for example, above the motor cortex. When a changing electric current flows through the coil, an electromagnetic field is created [1], [2]. According to Faraday's law, this induces an electric field in the brain that can stimulate cortical neurons [3]. The effects of TMS are often measured by behavioral observation, for example, involuntary, brief movement of the hand following stimulation over the motor cortex [4]. Thus, TMS differs from other noninvasive methods in that it can interfere with behavior, making it a powerful tool for investigating the relation between human behavior and brain activity.

TMS is characterized by many parameters: stimulus amplitude, pulse waveform, pulse duration, and the diameter and shape of the coil [5], [6]. The technique is commercially available and has been used in many cognitive psychology studies. Commercial magnetic stimulators use coils with an outer diameter of 50–150 mm and produce magnetic fields of 1–2.5 Tesla with a field rise time of 50–200 µsec [6]. Coil shapes, other than the ordinary round shape, have been developed to improve the behavioral response [7], [8].

Despite the wide use of TMS in cognitive research, the mechanism of neuronal excitation by TMS is largely unknown. To date there have been no direct recordings of the membrane potential from single neurons during a TMS pulse. Furthermore, most theoretical investigations of the TMS effect on single neurons have been limited to simple neurons. Most of the simulations have described the impact of magnetic stimulation (MS) on peripheral neurons, linear or bent, using either an analytical approach [9], [10], [11], [12], [13], [14] [15] or a numerical approach [16], [17], [18], [19], [20], [21]. Only one previous investigation has applied compartmental modeling to simulate the impact of magnetic stimulation on neurons with arbitrary morphology [22]. This study assumed that the induced electric field was spatially uniform and that the stimulus had a simple pulse shape. Thus, a complete description of the impact of MS on cortical neurons is still lacking, leaving important questions unanswered. What role does neuronal morphology play in MS? Which element of CNS neurons is most likely to be activated by MS? Will MS activate dendritic regenerative mechanisms? What is the magnetic threshold of different neurons in the cortex?

Here we attempt to address these questions by numerical modeling of the excitation of neurons in the central nervous system by a magnetic field. Clearly, the results of any modeling study should be subject to experimental verification. Our lab has substantial experience in recording from brain slices using the patch-clamp technique [23], [24], . Thus, to allow simpler transformation of the simulations into possible future in-vitro experiments, we simulated the case where a round coil is placed parallel to a neuron. The electric field induced by the magnetic field was calculated and integrated into a compartmental simulation using the simulation environment NEURON [29]. This allowed us to simulate the response of neurons with arbitrary morphologies to the magnetic field. We verified the accuracy of the simulation using simple neuronal structures and compared the results to previous studies [16], [17], [21]. We then demonstrated the effect of magnetic stimulation on simplified neurons and on full models of cortical neurons. Our simulations suggest that TMS differently activates CNS neurons and peripheral neurons. The largest impact on peripheral neurons was found at the location along the axon experiencing the largest gradient of the induced electric field. However, in CNS neurons, TMS was found to directly depolarize the soma, leading to initiation of an action potential (AP) in the initial segment of the axon.

## Results

### Implementing magnetic stimulation in NEURON

The magnetic pulse from a TMS device induces an electric field in the brain [30], which can generate transmembrane currents when it falls across the cell membrane of a neuron. However, as experimental studies have shown that neurons are insensitive to transverse field stimulation relative to axial stimulation [5], we neglected the electric field perpendicular to the membrane. The induced electric field can also generate an axial current when it interacts with the cytoplasmic resistor. This can be expressed as:
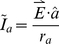
(1)where 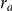
is the axial resistance per unit length, 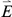 is the induced electric field, 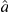 is a unit vector parallel to the axial direction of the segment. According to the passive cable theory, the additional membrane current caused by this axial current is:
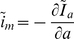
(2)Where *a* is the direction along the fiber. Inserting eqn. 1 into eqn. 2 we obtain [31]:
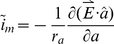
(3)


Multiplying eqn. 3 by the membrane resistance (r_m_) we obtain the induced change of the membrane potential:
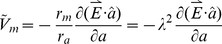
(4)where 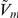
is the change in the membrane potential generated by the magnetic stimulation and λ is the passive space constant. This function, used to calculate membrane polarization due to changes in the external electric field, is known as the activating function [15], [16], [17], [19], [32], [33]_. _Eqn. 4 states that the strength of MS is determined by the directional derivative of the electric field along the segment direction [15] and by the intrinsic properties forming the passive space constant. From here, it is simple to derive the complete cable equation including the induced electric field [9], [10], [11], [12], [16], [17], [19], [34].
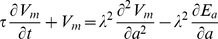
(5)where *V_m_* is the membrane potential, is the time constant, *a* is the direction along the fiber and *E_a_* is the projection of the electric field in that direction. Eqn. 5 has been successfully solved for several simple neuronal structures using analytical [11], [12] or numerical approaches [16], [17], [19], [22]. However, no study has yet solved Eqn. 5 for complex neurons using a realistic induced electric field within standard compartmental modeling software such as NEURON [29] or GENESIS [35]. To add the effect of the magnetically induced electric field to NEURON we used an approximation of Eqn. 3. Thus, given a short segment of the neuron defined by a starting point at 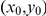 and an ending point at 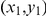 [36] we can approximate Eqn. 3 as a difference equation:
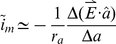
(6)


Furthermore, taking the center of the coil as the origin, the induced electric field in the direction of the neuronal segment can be represented using the Cartesian coordinate system. As we simulate the case in which a round coil is placed parallel to a neuron, we can neglect the changes in the magnetic field in the z direction. Thus, we can formulate the induced electric field for this case:
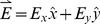
(7)and
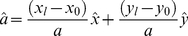
(8)


The dot product gives:
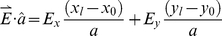
(9)


Thus, we obtain:
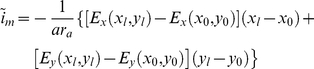
(10)


**Figure 1:**
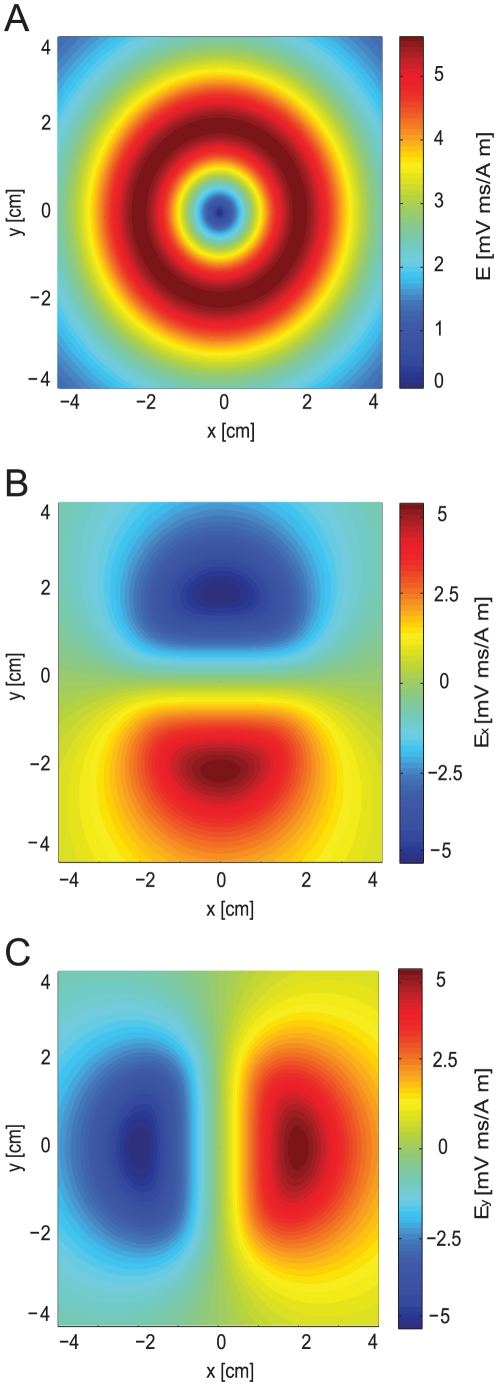
The induced electric field generated by the magnetic flux in a Cartesian coordinate system. The spatial part of the electric field was calculated in Matlab prior to the simulation with equation 18 and then exported from Matlab to NEURON. For simulation of peripheral neurons, matrix size was 80000×80000 µm with a spatial resolution of 1 µm. Distance from the plane of the coil was 1 cm, coil radius was 2 cm, 30 loops to the coil. The permeability constant was 4π*10^−7^ H/m. **A**, The spatial function of the induced electric field. **B**, The spatial component of the induced electric field along the x-axis. **C**, The spatial component of the induced electric field along the y-axis.

The additional membrane current induced by MS could be calculated in NEURON using equation 10. To simulate MS of a neuron with arbitrary morphology, this current was added to each segment of the neuron as a nonspecific leak current with a zero reversal potential, programmed using the NMODL extension of NEURON [29]. The Cartesian components of the electric field were calculated, assuming that the magnetic stimulation was generated by a circular coil that was part of an RLC circuit (eqns. 20–23). The spatial component (eqn. 18) of this induced electric field generated by the simulated RLC circuit is shown in Figure 1A. As described in the methods, the spatial component of the electric field (eqn. 18) was calculated in Matlab prior to the simulation and exported to NEURON as two matrices, one for E_x_ (Figure 1B) and one for E_y_, (Figure 1C) with a spatial resolution of 1 µm. The temporal component of the electric field (eqn. 19) was calculated in NEURON in every time step.

### Magnetic stimulation of a straight axon

The above generic numerical approach allows simulation of MS for arbitrary morphologies. However, to verify our numerical approach and observe the impact of MS on a simple neuronal structure we first simulated MS of a long straight axon. Numerical simulations of MS of long, straight axons have been performed using custom written code [16], [17], [19]. Here we examined the mechanism of stimulation using the activating function (eqn. 4). The magnitude of the membrane potential change is determined by the size of the gradient of the induced electric field and the passive space constant in the axon fiber (λ). Thus, for an axon fiber with spatially homogenous passive parameters, the major factor determining the shape of the membrane potential change, and thus the location of AP initiation during MS, is the gradient of the induced electric field while λ will act as a general scaling factor of the MS induced changes to the membrane potential.

**Figure 2:**
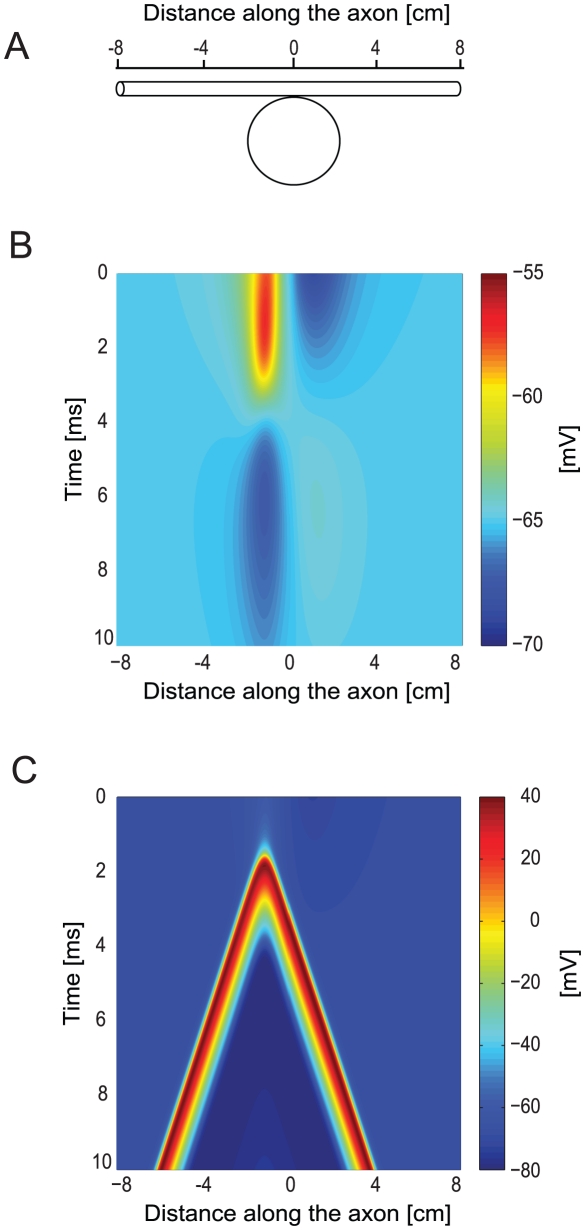
Magnetic stimulation of a peripheral axon. A straight axon was located in a plane below the coil and the coil was shifted along the y-axis by one coil radius. Distance from the plane of the coil was 1 cm, coil radius was 2 cm, 30 loops to the coil. The underdamped pulse was used (R = 0.09 Ω; L = 13 µH; C = 200 µF; τ = 0.4 ms). Axon diameter was 100 µm, length 16 cm. Magnetic threshold of the axon was 36 v. Nernst potentials, conductance, membrane capacitance and resistivity of axoplasm were all equal to NEURON's default of the Hodgkin-Huxley model. **A**, View of the system showing the magnetic coil from above and beneath it the straight axon (marked with scale). The coil was moved normal to the axon (y-axis). Here it is shown shifted away from the axon so that the coil's center lies at a distance of one coil radius from the axon. **B**, Membrane potential as a function of time and location along the axon for a subthreshold stimulus (V = 30 V). **C**, Membrane potential as a function of time and location along the axon for a suprathreshold stimulus (V = 36 V).

Similar to previous simulations [16], [17], a straight axon (diameter 100 µm, length 16 cm along the x-axis) was placed in a plane parallel to that of the coil (coil radius 2 cm, the axon lay 1 cm from the plane of the coil). As any change perpendicular to the axon (y-axis) is important for stimulating the axon [16], [17], [21], the coil was moved along the y-axis by one coil radius (Figure 2A). Under this configuration, the maximal induced electric field was located 1.6 cm from the coil center and not at the coil radius (2 cm) [16]. For this set of simulations we used the Hodgkin-Huxley model [37] with homogenous channel density throughout the axon membrane.

We first simulated the effect of subthreshold MS on the axon using an underdamped current pulse (eqn 22). The axon responded by hyperpolarization on one side of the coil and depolarization on the other side (Figure 2B). As the polarity of the current pulse changed the axon underwent smaller opposing depolarization and hyperpolarization, due to pulse behavior (Figure 2B). When MS amplitude was further increased, the depolarization induced by the stimulation was large enough to cross AP threshold. The two APs that were generated propagated along the axon in opposite directions (Figure 2C). The site of AP initiation corresponded to the location of the maximal gradient of the induced electric field. Since the axon fiber had spatially homogenous passive parameters, this location corresponded to that of the maximal activating function [33]. Both the subthreshold and suprathreshold responses of the axon obtained here agreed with previously published simulations of MS of straight axons [16], [17].

**Figure 3:**
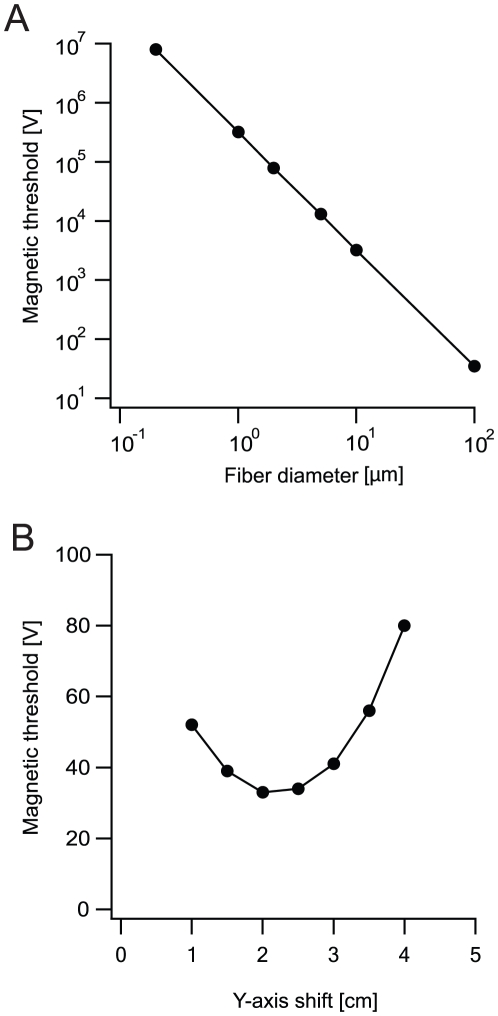
Factors affecting magnetic stimulation of peripheral axons. A straight axon was located in a plane below the coil and the coil was shifted along the y-axis by one coil radius. Distance from the plane of the coil was 1 cm, coil radius was 2 cm, 30 loops to the coil. The underdamped pulse was used (R = 0.09 Ω; L = 13 µH; C = 200 µF; τ = 0.4 ms). Nernst potentials, conductances, membrane capacitance and resistivity of axoplasm were all equal to NEURON's default of the Hodgkin-Huxley model. **A**, Magnetic threshold as a function of fiber diameter shown on a log-log scale. The line is a curve fit of V_th_ = a+b/d^2^ where V_th_ is the magnetic threshold, a and b are proportionality constants and d is the fiber diameter **B**, Magnetic threshold as a function of shifting the coil along the y-axis. Distance from the axon was measured with respect to the center of the coil. Axon diameter was 100 µm.

To further investigate the effects of MS on straight axons we examined the impact of various parameters on the ability of MS to generate an AP. First, we determined the minimal intensity of MS required to generate an AP similar to that displayed in figure 2C. MS was simulated several times, increasing the voltage applied to the simulated coil until an AP was generated. The threshold for AP generation by an electromagnetic pulse is thus given in volts applied to the RLC circuit and is referred to as the magnetic threshold [10]. As predicted by eqn. 4 and similar to previous simulations [11], [17], [19], increasing the diameter of the axon decreased the magnetic threshold (Figure 3A), which was proportional to the inverse square of the axon diameter (Figure 3A) [11], [17], [19]. Shifting the coil relative to the axon altered the magnetic threshold (Figure 3B). The lowest magnetic threshold was obtained when the coil was shifted relative to the axon by one coil radius (Figure 3B).

### The impact of basic neuronal morphology on magnetic stimulation

When a structurally homogenous axon is longer than the coil radius, the gradient of the induced electric field creates membrane potential gradients along the fiber. In contrast, CNS neurons are likely to be shorter than the radius of the coil in a standard TMS setup. Thus, a neuron may experience different gradients of the induced electric field depending on its location relative to the coil. The complex morphology of CNS neurons may cause further differences. Eqn. 4 predicts that changes in the electrotonic structure of the neuron modify the neuron's response to MS. Changes in the diameter of the fiber, the axial resistance and membrane resistance lead to changes in the space constant (λ) of the neuron. Such changes are expected to alter the contribution of the induced electric field to the membrane potential of a given compartment. Furthermore, changes in the diameter or orientation of the neuron has been predicted to affect the neuronal response to MS [22].

**Figure 4 pcbi-1002022-g004:**
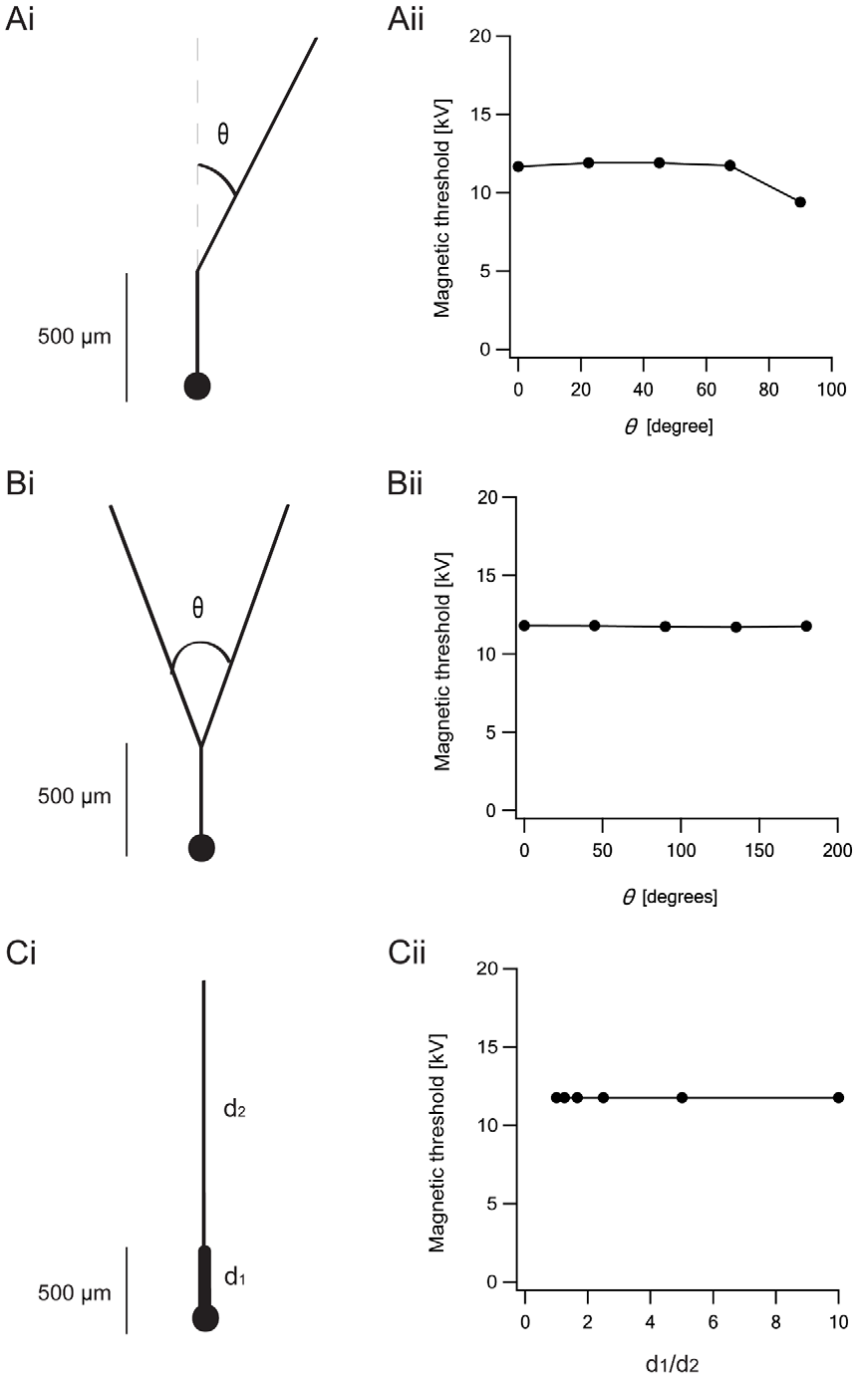
Basic neuronal structures affect magnetic stimulation. The artificial neurons were located at the center of the matrices as demonstrated with a pyramidal cell in Figure 6. Distance from the plane of the coil was 1 cm, coil radius was 2 cm, 30 loops to the coil. The underdamped pulse was used (R = 0.09 Ω; L = 13 µH; C = 200 µF; τ = 0.4 ms). The artificial neurons contained a soma simulated with the Hodgkin-Huxley model to generate neuronal excitability, while the dendrites contained passive parameters. The diameters of the soma and dendrites were 20 µm and 5 µm respectively, with the exception of dendrites in the bifurcation structure, whose diameter was set at 3.1498 µm. This was calculated according to the d^3/2^ law developed by Rall [60], [61], [62]. **Ai**, The dendrite had a bend at one point along its length. θ is the angle between the second dendrite and the imaginary continuation of the first dendrite. **Aii**, The magnetic threshold as a function of θ for the bent dendrite. **Bi**, The primary dendrite bifurcated into two branches with equal diameter. Here, θ is defined as the angle between the second and the third dendrite. **Bii**, The magnetic threshold as a function of θ for the bifurcating dendrite. **Ci**, A cell with a change in dendrite diameter. **Cii**, The magnetic threshold as a function of the ratio of the diameters of the first to the second dendrite segment.

We investigated the effect of MS on several simplified structures commonly found in CNS neurons. These were a bend, a bifurcation, and a diameter change, all of which may be located at different positions in the dendritic and axonal trees of CNS neurons. We investigated the case where a neuron contained an excitable soma and passive dendrites. We generated simplified artificial neurons containing a soma into which we inserted the Hodgkin-Huxley model to generate neuronal excitability. A single passive dendrite originated from this soma. This dendrite was allowed to bend at one point along its length (Figure 4Ai), to bifurcate (Figure 4Bi), or to change diameter at a single point (Figure 4Ci). The angle of the bend was changed systematically.

**Figure 5:**
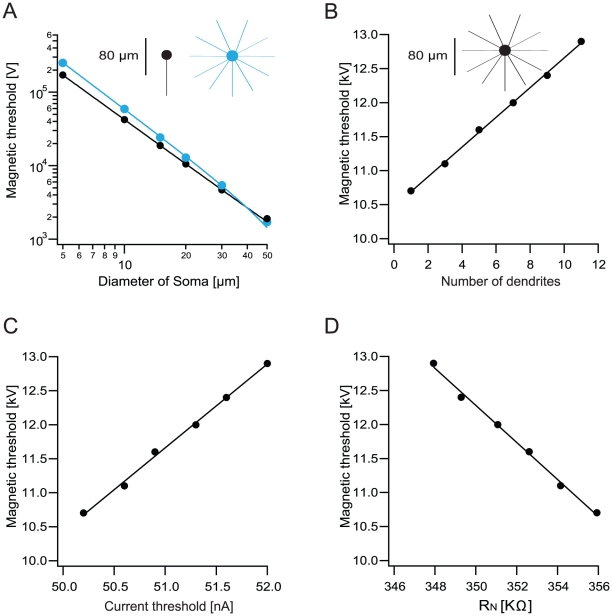
Dendrites act as current sinks modifying magnetic threshold. **A**, Magnetic threshold as a function of soma diameter for a soma with one dendrite (black) and a soma with 11 dendrites (light blue). The lines is a curve fit of V_th_ = a+b/d^2^ where V_th_ is the magnetic threshold, a and b are proportionality constants and d is the fiber diameter. **B**, Magnetic threshold as a function of the number of dendrites. The number of dendrites connected to the soma was increased and the magnetic threshold plotted for every cell. A soma with 6 dendrites is shown in the insert as an example. **C**, Magnetic threshold as a function of current threshold for cells with different numbers of dendrites. **D**, Magnetic threshold as a function of input resistance for cells with different numbers of dendrites. Location of the artificial cell with respect to the center of the coil and the parameters used for calculating the induced electric field as in figure 4. Soma and dendrite diameters were 20 µm and 1 µm, respectively.

The magnetic threshold decreased when the bend angle increased above 70°(Figure 4Aii). Modifying the angle of the bifurcation (Figure 4Bii) or the diameter at one location along the dendrite (Figure 4Cii) did not change the magnetic threshold. Conversely, increasing the diameter of the soma dramatically reduced the magnetic threshold for two artificial neurons – one, a soma with a single dendrite (black) and, the second, a soma with 11 dendrites (light blue, Figure 5A). As with the diameter of the peripheral axon (Figure 3A), the magnetic threshold was proportional to the inverse square of the somatic diameter.

The simulations presented in Figures 4, 5A suggest that in neurons smaller than the coil radius, the MS acts on the soma, while the dendrites remain relatively unaffected. To further investigate the role of the dendrites we progressively added passive dendrites to the soma (Figure 5B insert). As the number of dendrites increased, so did the magnetic threshold (Figure 5B), each added dendrite serving as a current sink drawing current from the soma. To substantiate this explanation we measured the current threshold for the same cells as a function of the number of dendrites projecting from the soma. To do this we simulated a current-clamp electrode placed at the soma through which a square current pulse was injected. The amplitude of this current pulse was increased until an AP was generated. The current threshold obtained was highly correlated with the magnetic threshold (Figure 5C). An increase in the somatic input resistance generated a decrease in magnetic threshold (Figure 5D). All the changes to the magnetic threshold induced by the number of passive dendrites were substantially smaller than those induced by changing the diameter of the soma (Figure 5A).

The simulations presented in Figures 3–5 suggest a mechanism for excitation of neurons smaller than the radius of the magnetic coil. In agreement with the activating function (eqn. 4), the compartment with the largest diameter (i.e. the soma) and, therefore, the largest passive space constant, undergoes the largest depolarization. The larger the diameter of the segment, the larger will be the depolarization for a given stimulation (Figs. 3A, 5A), leading to a lower magnetic threshold. This depolarization is attenuated by current escape into the dendrites, which, because of their smaller diameter, are less affected by the magnetic pulse.

### Cells with realistic morphology

**Figure 6:**
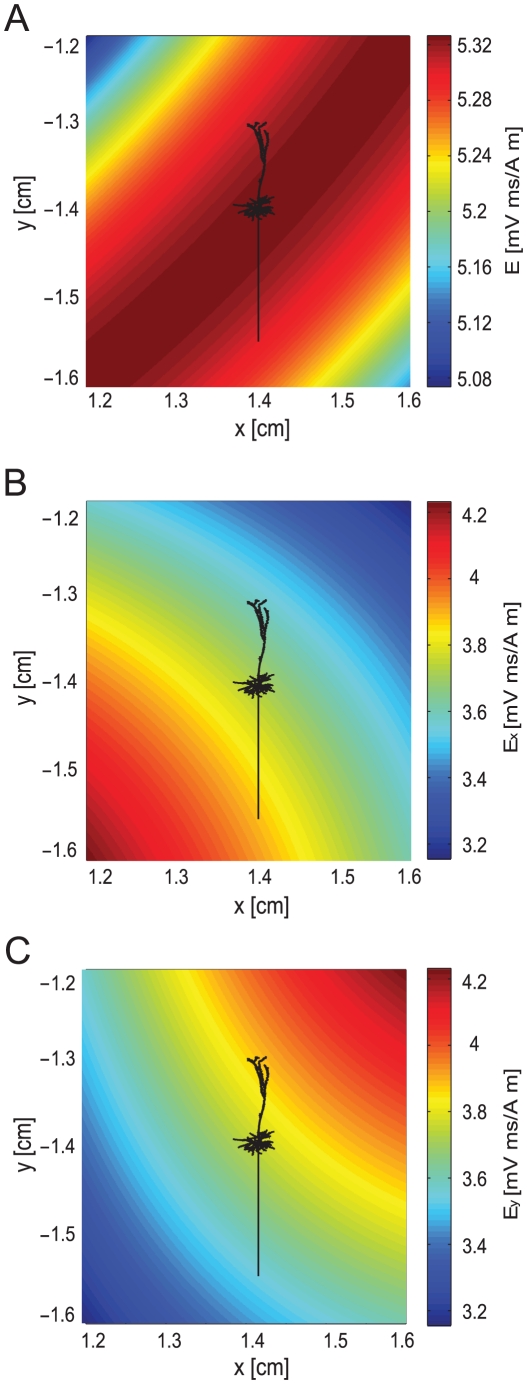
The induced electric field generated by the magnetic flux in a Cartesian coordinate system. The spatial part of the electric field was calculated in Matlab prior to simulation with Equation 18 and then exported from Matlab to NEURON. For the simulation of CNS neurons, matrix size was 4000×4000 µm with a spatial resolution of 1 µm. The center of the matrix field lay 1.98 cm from the center of the coil. The size relation between the matrices and a neuron is demonstrated by a pyramidal neuron located in the center of the field. Distance from the plane of the coil was 1 cm, coil radius was 2 cm, 30 loops to the coil. The permeability constant was 4π*10^−7^ H/m. **A**, The spatial function of the induced electric field. **B**, The spatial component of the induced electric field along the x-axis. **C**, The spatial component of the induced electric field along the y-axis.

The primary output neurons of the cortex are the large pyramidal neurons from layer 5. The dendritic trees of these neurons span all the layers of the cortex and their axons extend into the white matter. These features make L5 pyramidal neurons primary targets for MS [15]. To fit the simulation to CNS neurons without overtaxing the memory of the computer, the spatial part of the induced electric field was represented as a 4000X4000 µm matrix with 1 µm resolution (Figure 6A). As described above and in the methods, the spatial part of the electric field (eqn. 18) was calculated in Matlab prior to the simulation. It was exported to NEURON as two matrices, one for E_x_ (Figure 6B) and one for E_y_, (Figure 6C) with a spatial resolution of 1 µm. Note that the color scale in figure 6 spans only a fraction of the same scale in figure 1. Figure 6 shows the size of pyramidal neuron compared to the induced electric field where the soma was placed 1.4 cm from the coil's center (Figure 6A). The excitability of L5 pyramidal neurons has been modeled in many studies. Here we used two established compartmental models of these cells [27], [38]. In addition to AP initiation in the axon initial segment, both models feature active back-propagation of the AP into the dendritic tree and generation of dendritic calcium spikes in the distal apical dendrite.

**Figure 7 pcbi-1002022-g007:**
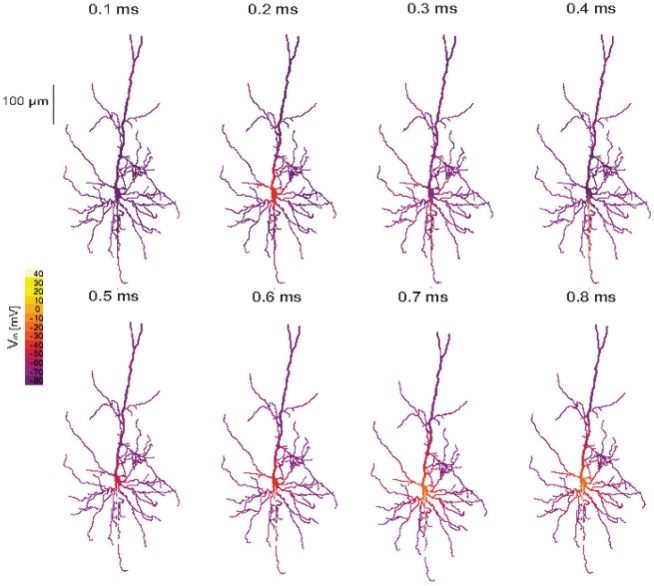
Magnetic stimulation stimulates the somato-axonal compartment in realistic neurons. The suprathreshold activity of a pyramidal neuron cell exposed to magnetic stimulation. The neuron was located at the center of the matrix as in Figure 4. Distance from the plane of the coil was 1 cm, coil radius was 2 cm, 30 loops to the coil. The underdamped pulse was used (R = 0.09 Ω; L = 13 µH; C = 200 µF; τ = 0.4 ms). Excitability was added to all cells using a model of neocortical pyramidal neurons [27]. The membrane potential (in mV) along a pyramidal neuron is displayed as pseudo-color in each compartment and several time points following MS initiation are shown in a time-lapse sequence.

Both models responded similarly to MS. Figure 7 shows the spatio-temporal response of one of these models [27] to a threshold underdamped MS. The initial impact of the MS was visible 0.2 ms after stimulus onset as a depolarization of the soma. Then, due to the oscillating nature of the underdamped waveform, the soma hyperpolarized (0.3 ms) and then depolarized again (0.4 ms). Because of the lower AP threshold in the axon, the initial somatic depolarization generated an AP in the axon initial segment 0.3 ms after the onset of the MS. This AP then back-propagated into the soma and dendrites and forward-propagated into the axon generating additional APs at the nodes of Ranvier along the axon.

**Figure 8 pcbi-1002022-g008:**
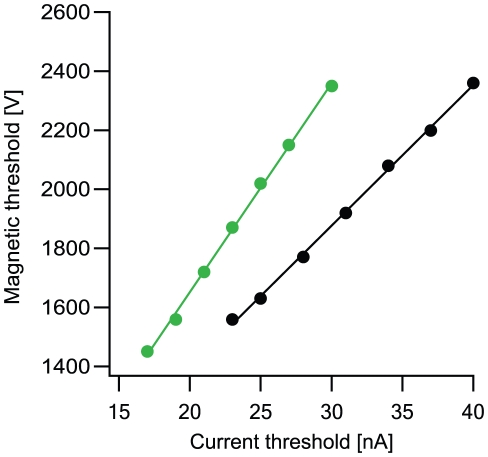
Magnetic threshold correlates with current threshold in realistic morphologies. Neurons were located at the center of the matrix as in Figure 4. Distance from the plane of the coil was 1 cm, coil radius was 2 cm, 30 loops to the coil. The underdamped pulse was used (R = 0.09 Ω; L = 13 µH; C = 200 µF; τ = 0.4 ms). Excitability was added to all cells using two different models of L5 pyramidal neurons - Larkum et al.'s model (2009) [38] (green) and Schaefer et al.'s model (2003) [27] (black). The magnetic threshold correlated with current threshold in pyramidal cell with different current thresholds. Sodium channel activation and inactivation were shifted towards hyperpolarizing potentials to reduce current threshold.

To investigate the contribution of cellular excitability to the response of CNS neurons to MS, the activation and inactivation curves of the voltage-gated sodium channels in the axon initial segment and nodes of Ranvier were shifted to more negative potentials. This reduced the current threshold as measured by somatic current injections. The magnetic threshold was linearly correlated to the current threshold also in this experiment (Figure 8). This suggests that low threshold neurons in the CNS may respond to lower intensities of MS.

**Figure 9 pcbi-1002022-g009:**
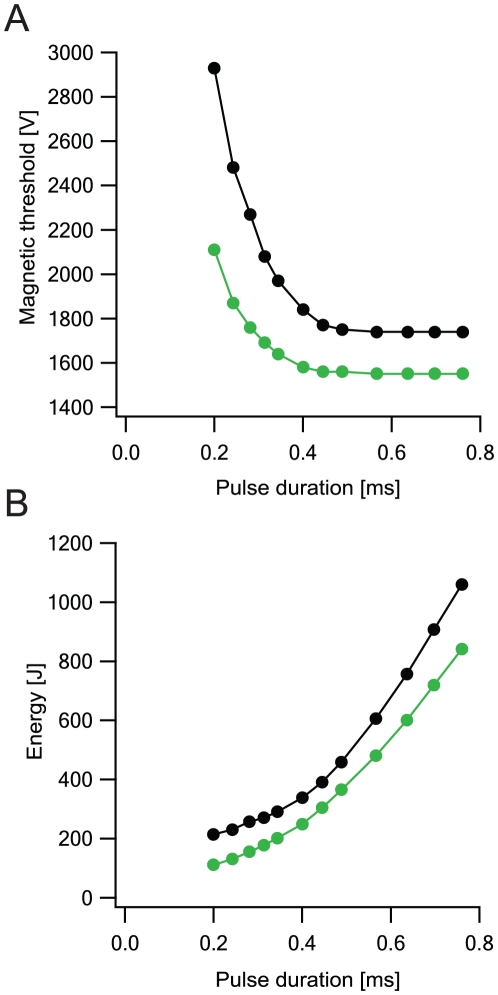
Magnetic threshold changes with pulse duration in realistic morphologies. Neurons were located at the center of the matrix as in Figure 4. Distance from the plane of the coil was 1 cm, coil radius was 2 cm, 30 loops to the coil. The underdamped pulse was used (R = 0.09 Ω; L = 13 µH; C = 200 µF; τ = 0.4 ms). Excitability was added to all cells using two different models of L5 pyramidal neurons - Larkum et al.'s model (2009) [38] (green) and Schaefer et al.'s model (2003) [27] (black). **A**, Magnetic threshold as a function of pulse duration (strength-duration curve). Pulse duration modified by changing the capacity from 50 µF to 700 µF. **B**, The device energy as a function of pulse duration.

Commercial TMS devices commonly generate a 0.4 ms underdamped pulse. We used our simulated model of L5 pyramidal neurons to investigate whether this pulse selection is optimal. We varied the duration of the underdamped pulse by changing the capacitance in the RLC circuit and measured the magnetic threshold for AP generation (Figure 9A). As pulse duration increased, the magnetic threshold decreased, resembling a strength-duration curve. This result suggests that it may be better to use longer stimulation times in TMS devices, since this allows the use of weaker magnetic fields. However, it is important to note that increasing pulse duration by increasing the capacitance requires that the MS device generate more energy (Figure 9B) [13]. This higher energy requirement may pose an engineering limit to TMS devices. The optimal time range predicted by figure 9 falls close to that used by commercial TMS devices.

**Figure 10 pcbi-1002022-g010:**
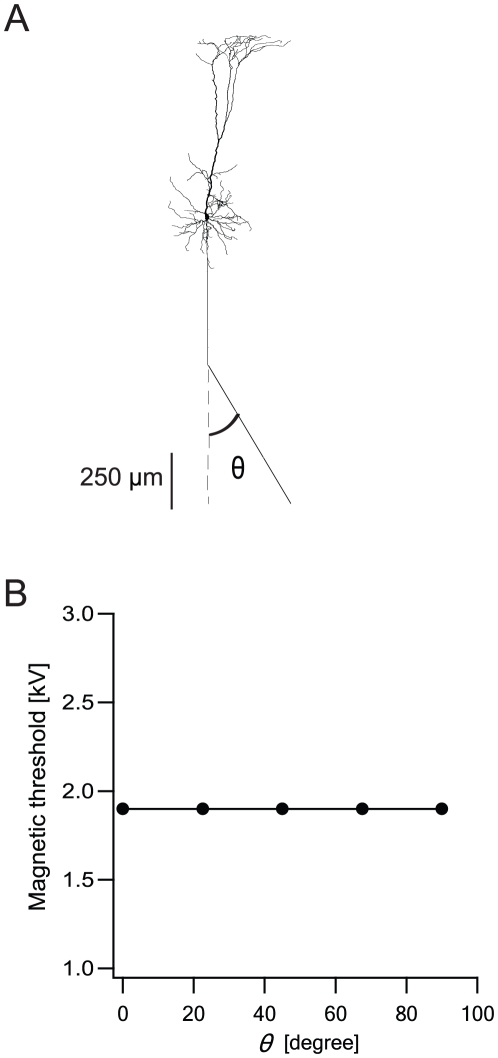
Magnetic threshold does not change with different angles of a bent axon. Neuron was located at the center of the matrix as in Figure 4. Distance from the plane of the coil was 1 cm, coil radius was 2 cm, 30 loops to the coil. The underdamped pulse was used (R = 0.09 Ω; L = 13 µH; C = 200 µF; τ = 0.4 ms). Excitability was added to all cells using a model of neocortical pyramidal neurons [27]. **A**, The pyramidal neuron with the bent axon. θ is the angle between the axon and its imaginary continuation. **B**, The magnetic threshold as a function of θ.

It has been suggested that a likely site for MS to cause AP generation in neocortical pyramidal neurons is where their axons bend as they enter the white matter [39]. This may be an additional mechanism by which MS generates APs in cortical neurons. To investigate this suggestion we performed numerical simulation on a full model of a neocortical pyramidal neuron and introduced a bend in the axon (Figure 10A). Contrary to the previous suggestion, this bend did not change the magnetic threshold at all (Figure 10B). In all simulations the AP was generated at the axonal initial segment following somatic depolarization. Although the bend in the axon did induce a larger depolarization at the bend, it was too small to generate an AP in any of the nodes of Ranvier along the axon.

**Figure 11 pcbi-1002022-g011:**
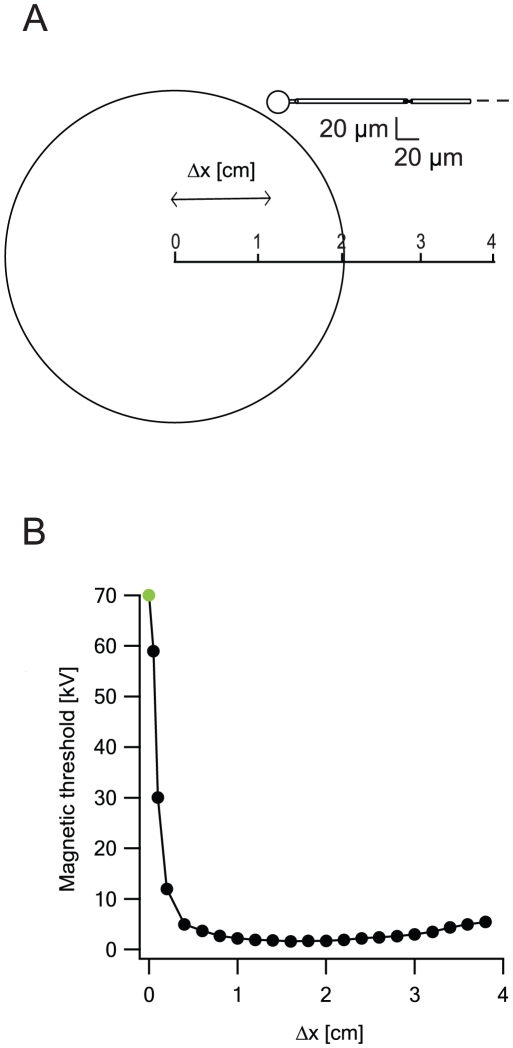
The location of action potential initiation and the magnetic threshold depend on the location of the soma relative to the coil. **A**, A soma with a long straight axon containing sections of myelin and nodes of Ranviar was located in a plane below the coil. Soma, axon and node diameters were 20 µm, 1 µm and 0.75 µm, respectively. Soma, axon and nodes lengths were 20 µm, 100 µm and 1 µm, respectively. The view from above shows that the artificial neuron was shifted along the y-axis by one coil radius and along the x-axis by Δx. **B**, The magnetic threshold decreased with Δx until it reached a minimum at the location corresponding to the maximal gradient of the electric field. The action potential was initiated at the axon for small shifts (Δx<0.05 cm, green dots), and at the soma for larger shifts (black dots). The lowest magnetic threshold was achieved when the soma was located at the largest gradient of the induced electric field.

To further emphasize the distinction between MS of peripheral and cortical neurons we investigated the case shown in figure 11A. A soma with a long myelinated axon was first located parallel to the center of the coil with a shift in the y-axis equal to one coil radius. The artificial neuron was then shifted along the x-axis and the magnetic threshold measured for each location (Figure 11B). When the soma was close to the vertical midline of the coil (Δx<0.05 cm, marked in green), the maximal activating function was located c. 1.6 cm from the center of the coil. This location underwent the largest depolarization and was where a suprathreshold stimulus initiated an action potential. As the soma was moved away from the center of the coil, the location of the maximum activating function, and hence the maximum depolarization, shifted to the soma. As predicted from eq. 4, the lowest magnetic threshold was achieved when the soma, which has the largest λ, was located where the gradient of the induced electric field is largest. This figure suggests that, while an AP can be initiated in the axon of CNS neurons, AP initiation is more likely at the soma and will occur there at lower MS intensities.

**Figure 12 pcbi-1002022-g012:**
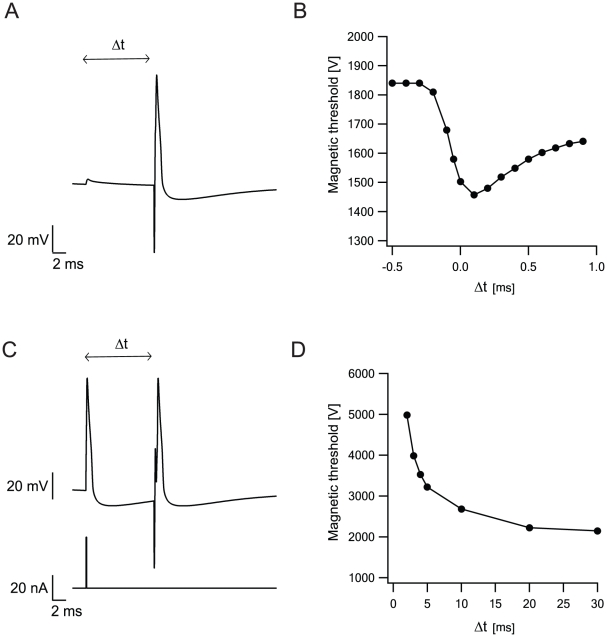
Neuronal activity modulates magnetic threshold. The effect of a change in membrane potential on the MS of the pyramidal cell model used in figure 8. Location of the artificial cell relative to the center of the coil and the parameters for calculating the induced electric field as in figure 5. Cell parameters as in figure 7. **A**, Magnetic threshold as a function of a synaptic input Δt before stimulus onset. **B**, The membrane potential in the soma as a function of time. An excitatory postsynaptic potential was evoked Δt milliseconds before MS onset. **C**, Magnetic threshold as a function of an AP at Δt before stimulus onset. **D**, Membrane potential in the soma as a function of time. An AP was evoked Δt milliseconds before MS onset.

The cell membrane is rarely at rest. Excitatory or inhibitory postsynaptic potentials (EPSPs, IPSPs) increase or decrease the membrane potential, respectively. A depolarization would be expected to reduce magnetic threshold for it is easier to stimulate a cell closer to its AP threshold. We tested this by evoking an EPSP in the cell a few milliseconds before the magnetic stimulus (Figure 12A). The magnetic threshold decreased, when the EPSP coincided with the MS (Figure 12B). We also investigated the impact of AP generation on the response of the simulated neuron to MS using an AP evoked before the magnetic stimulus (Figure 12C). In the models used for the current simulation there is a pronounced afterhyperpolarization lasting several tens of milliseconds after the AP. This period is often referred to as the refractory period. During this period an AP is harder to obtain due to the inactivation of sodium channels and activation of potassium channels. Figure 12D shows the magnetic threshold as a function of the time interval between the AP and MS. As predicted, the magnetic threshold increased the closer in time AP initiation was to the MS (Fig 12D).

The dendrites of L5 pyramidal neurons, and of many other cortical neurons, are not passive. Action potentials initiated at or near the soma actively back-propagate into the dendritic tree [40]. Furthermore, the distal apical dendrite of L5 pyramidal neurons generates complex regenerative Ca^2+^ and Na^+^ spikes [36], [41], [42], [43], [44], [45]. In these neurons, when a back-propagating action potential coincides with distal synaptic input, a dendritic Ca^2+^ spike is generated, leading to the generation of a burst of action potentials at the soma [36]. Under some conditions, Ca^2+^ spikes can be isolated in the dendrites, while under others, the spikes can spread to the soma [42], [43], [46], [47], [48]. Could MS generate dendritic Ca^2+^ spikes in compartmental models of L5 pyramidal neurons?

**Figure 13:**
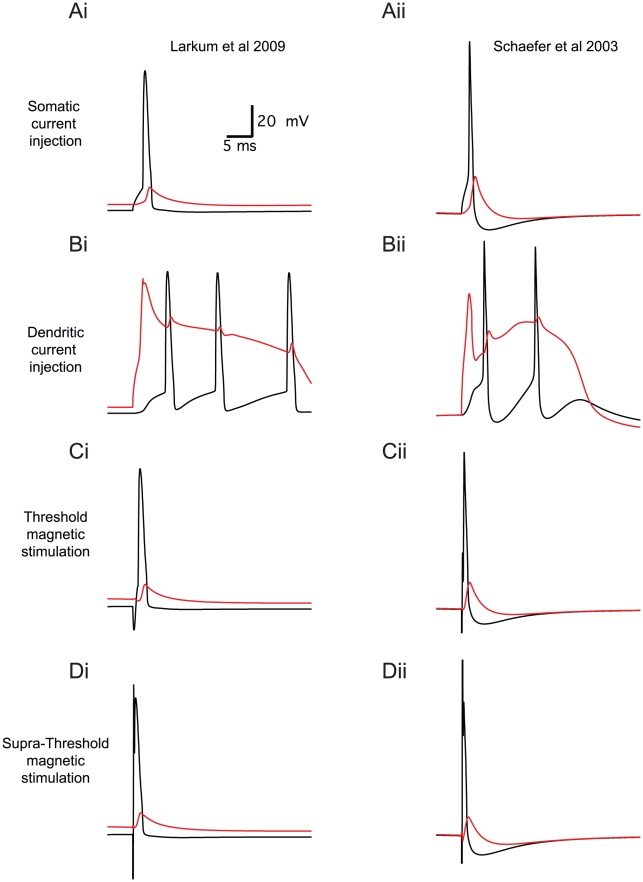
Magnetic stimulation generates axonal firing in pyramidal neuron models but not dendritic calcium spikes. Simulations of the effect of intracellular current injection and magnetic stimulation on somatic and dendritic membrane potential in two different models of L5 pyramidal neurons. Somatic membrane potential is shown in black and the dendritic membrane potential (600 µm along the apical dendrite) in red. The left column of the figure displays the response of the Larkum et al. model (2009) [38]. The right column of the figure displays the response of the Schaefer et al. model (2003) [27]. A, The response of the models to a 2 ms somatic current injection. B, Response of the model to a 2 ms dendrite current injection (at 600 µm along the apical dendrite). C, Response of the models to a 400 µs threshold MS. D, Response of the models to 400 µs MSs five times stronger than the threshold stimulation in C.

Both models used in this study can generate dendritic Ca^2+^ spikes. When a 2 ms current pulse was injected into the soma of these models, an AP was generated in the axonal initial segment (Figure 13A, black line). This AP actively back-propagated to the apical dendrite (Figure 13A, red line). Injecting a similar current pulse into the apical dendrite approximately 600 µm away from the soma caused generation of a complex Na^+^-Ca^2+^ spike (Figure 13B). This dendritic spike induced the firing of a burst of APs at the axon initial segment. Threshold MS of the model neurons generated an AP at the axon initial segment (Figure 13C). The back-propagation was similar to that observed with a somatic current injection. Increasing the MS to 5 times the magnetic threshold still generated an AP at the axon initial segment. Even at considerably higher MS intensities we were not able to generate a dendritic Ca^2+^ spike using MS (Figure 13D).

## Discussion

We have presented a numerical scheme allowing computation of the effect of magnetic stimulation (MS) on neurons with arbitrary morphology. By combining numerical simulations in Matlab and NEURON we were able to calculate the induced electric field for any arbitrary coil geometry and stimulus shape. We validated this approach by comparing our simulations to previous simulations of the impact of MS on peripheral neurons [9], [10], [11], [12], [13], [14], [16], [17], [18], [19], [20]. We then simulated the effect of MS on simple neuronal structures. These simulations suggested that the soma of CNS neurons is the primary locus of their response to MS. Finally, we simulated MS using realistic morphologies. Our simulations give rise to several predictions useful for designing electrophysiological experiments both *in vitro* and *in vivo.*

Several assumptions were made during the construction of the numerical simulation scheme. First, we assumed that only the component of the electric field parallel to the neuronal compartment was responsible for neuronal excitation, as in other simulations of MS of peripheral and central neurons [10], [11], [12], [16], [17], [49]. Thus, the induced electric field induced currents in the axial resistor, the cytoplasm. Current flowing axially in the cytoplasm is linked by passive cable theory to the membrane current (eqn. 4). The component of the induced electric field that is normal to the membrane alters the membrane potential, expressed as 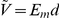, where *E_m_* is the electric field in the direction of the membrane (normal to the direction of the segment) and *d* is the thickness of the membrane. Since the membrane is about 3–10 nm thick, the contribution of the perpendicular electric field is negligible. Alternatively, *d* can be viewed as the combined thickness of the cytoplasm and the membrane [21]. In this case the normal component of the electric field generates a larger current [21]. However, in thin CNS dendrites and axons even this current will still be much smaller than the axial current. Furthermore, for a neuron lying in a plane parallel to the coil, symmetry can also be taken into account. The perpendicular electric field hyperpolarizes one side of the cell, while the other side is depolarized, the symmetry canceling out the overall change [5]. Some experimental evidence from stimulating the human median nerve suggests that the contribution of the electric field normal to the membrane is not negligible [34]. However, the median nerve inside a human arm does not run entirely parallel to the coil, which may cause problems in separating the normal from the parallel component of the electric field. This observation requires further investigation with *in vitro* studies. A second assumption was that the neuron is two dimensional, lying in a plane parallel to the coil. This allowed us to neglect calculating the decay in the magnetic field as a function of distance from the coil. This assumption simplifies the calculation of the induced electric field at the cost of potential errors due to the three-dimensional structure of the neuron. Obviously, the validity of this assumption must be reassessed for simulations of MS in whole brain tissue.

To validate our numerical approach we repeated several simulations of the effect of MS on peripheral axons. All the results (Figures 2–3) resembled previously published simulations [11], [12], [16], [17]. The similarity extended to the temporal and spatial pattern of excitability in the axon (Figure 2C), to the location of AP initiation relative to the coil (Figure 2C) [50], to the relationship between the position of the coil and the magnetic threshold (Figure 3B) as previously suggested [20], [34], and to the relationship between the axon diameter and the magnetic threshold (Figure 3A) that was previously simulated [17]. Our results conflict with a minority of the published simulations which claim that the strongest stimulation occurs at the maximum of the electric field, meaning, in this case, the middle of the fiber [51].

It has been previously suggested, both theoretically [22] and experimentally [10], that the magnetic threshold for the generation of an AP is dependant on neuronal morphology. Uniform electric fields have been suggested, both theoretically [39] and experimentally [52], to stimulate somata of CNS neurons. In addition, it was proposed that bends in the axons of cortical neurons might be possible locations for AP generation during stimulation with uniform electric fields [39]. Experiments on frog sciatic nerve have shown that MS can excite a nerve only where there are endings or where the nerve course curves, the curvature magnifying the effect of the magnetic field [10]. Compartmental modeling has suggested that an AP is generated only if there is a difference between the influence of the stimulation on adjacent compartments [22].

Our simulations here suggest that the impact of bends, bifurcations and diameter changes is secondary to changes in the diameter of the soma (cf. figs 4, 5A, 10). For neurons smaller than the radius of the magnetic coil, the simulations presented in figures 4, 5 show that the compartment with the largest diameter (i.e. the soma) undergoes the largest depolarization. This result can be directly extracted from the activating function (eqn. 4). Given homogenous passive parameters and a relatively shallow electric field gradient, the major difference between the soma and the other compartments in the neuron is their diameter. Since the effect of the induced electric field is scaled in eqn. 4 by the passive space constant, it is largest at the soma. This somatic depolarization is attenuated by current escape into the dendrites that are less affected by the magnetic pulse due to their smaller diameter (Figure 5A). For example, with a soma of 20 µm diameter and a dendrite 2 µm in diameter, MS induces 100 times more depolarization at the soma. This ratio is even larger for sub-micron axons and nodes. Note that MS of long peripheral neurons follows a different mechanism since the axons are longer than the coil radius and excitation is obtained using the mechanism presented in figure 2, as already described in the literature [11], [12], [16], [17], [21]. This difference in possible excitation mechanisms between peripheral and central neurons warrants caution when interpreting results of TMS of the central nervous system using stimulation rules based on MS of peripheral neurons.

It is interesting to compare MS to stimulation of CNS neurons using an extracellular microelectrode, the latter case having undergone considerable investigation [32], [33], [53]. It is clear that both MS and electrical microstimulation induce an electric field in the brain tissue that stimulates neuronal elements according to equation 4. Therefore the major difference between the two stimulation methods does not stem from physical principles but from geometrical ones. Microstimulation generates a spherical electric field that is maximal at the electrode and decays as a function of distance. That is, microstimulation has the largest impact close to the electrode, depending on the excitability of adjacent neural elements, the strength of the stimulation and the orientation of the induced electric field to the neural element [32], [33], [53]. Indeed, microstimulation has been shown in countless reports to stimulate all types of excitable compartments in the CNS – dendrites, somata and axons. Neural elements with lower excitation threshold that are further away from the stimulating electrode are not stimulated due to the decay of the electric field.

It is harder to verbally describe the distributed geometry of the electric field induced in brain tissue by a magnetic coil. Basically, the most striking difference between this field and that induced by a microelectrode is its spatial distribution. Since MS coils are usually a few centimeters in diameter the induced electric field spans much larger areas of the brain. Thus, it first excites neural elements with low excitation threshold and may lead to generation of AP at the axon initial segment due to somatic depolarization. Furthermore, our simulations (Figure 8) suggest that neurons with low current threshold, such as inhibitory interneurons, will be stimulated at lower MS intensities. This notion of initiating cells has been proposed recently following imaging experiments during MS in tissue cultures [49]. In conclusion, we predict that, within the same brain region, microstimulation and MS will stimulate different populations of neurons.

Simulating MS of realistic neuronal models (Figs 6–13), we observed similar trends to those obtained using artificial neurons. The simulations tightly linked the current threshold for AP firing to the magnetic threshold, further indicating that the soma is the primary element in the neuron's response to a magnetic stimulus. The current threshold could be modified by using dendrites as current sinks (Figure 5) or by shifting the activation kinetics of the voltage-gated sodium channel (Figure 8). These simulations lead to a prediction that can be investigated experimentally. As various classes of cortical neurons display either low or high current thresholds, we predict that the current threshold, measured using intracellular recordings from neurons in brain slices or tissue culture, is correlated with the magnetic threshold of these neurons. As some inhibitory cortical interneurons are known to have low current thresholds [54], [55], it is tempting to speculate that these neurons will also have a low magnetic threshold. If this is the case, it is even more tempting to hypothesize that low intensity TMS may selectively activate inhibitory cortical interneurons, while higher intensity TMS may activate both inhibitory and excitatory neurons. Our simulations also predict that the magnetic threshold will fall when excitatory synaptic activity coincides with MS (Figure 12A) and rise when an AP is generated coincident with MS (Figure 12B). Furthermore, our simulations predict that dendritic calcium spikes are not activated directly by MS (Figure 13).

A recent imaging experiment with primary cultures of hippocampus neurons has provided some support for the relationship between magnetic threshold and intrinsic neuronal excitability [49]. This elegant study reported that a small group of neurons responded with higher sensitivity to MS. Possibly, some of the neurons from the hippocampal culture had lower electric, and therefore lower magnetic, thresholds. Since the study recorded only the intracellular calcium concentration, it was possible only to observe the excitation of the neuron and not its current threshold. Another recent study, stimulating neurons in brain slices by uniform electric fields, has shown that neuronal morphology correlated with somatic subthreshold deflection of the membrane potential [52]. This study also observed, in agreement with our predictions, larger somatic depolarization in L5 pyramidal neurons than in interneurons with smaller somata.

Some predictions arising from our work can be verified using intracellular recordings of membrane potential, for example the correlation between the magnetic threshold and the current threshold (Figure 8A). Magnetic threshold for different cell types can be measured and compared to our theoretical predictions. Furthermore, intracellular recordings may verify our predicted effect of synaptic input or AP generation on magnetic threshold (Fig 12) and the preferred activation of the axon initial segment over dendritic spikes in pyramidal neurons (Figure 13). Simultaneous recordings of the membrane potential from the soma and dendrites [23], [24], [25], [26], [27], [28] can test our prediction that MS induces the largest depolarization at the soma. Moreover, simultaneous recordings from soma and axon during MS should also indicate which is excited first. Finally, combining our simulation environment with intracellular recordings will allow probing many features of MS (including pulse shape, pulse duration, coil shape and properties, etc.) currently unavailable in commercial TMS devices, eventually leading to the design of improved TMS devices and stimulation protocols.

## Methods

### Simulation environment and compartmental models

A magnetic field is generated when an electric current is passed through a magnetic coil. This magnetic field can be presented as the curl of the vector potential (A):
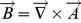
(11)


The magnetic field induces an electric field in the neural tissue that is composed of the electric scalar potential (V) and the magnetic vector potential (A) [56]: 
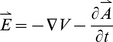
(12)


Assuming that there is no charge accumulation, then the electrical scalar potential is negligible [10], [18], [56]. The vector potential is affected by the geometry of the coil, the number of loops in the coil, and the electric current running through the coil [18]: 
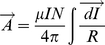
(13)where 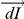 is a current element, R is the distance from the current element, N is the number of loops in the coil, I is the current through the coil and µ is the permeability constant. Given a round coil, the general solution is:
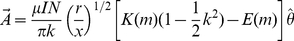
(14)

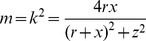
(15)where 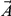 is the vector potential, *r* is the coil radius, *x* is the distance of the point from the center of the coil and K(m) and E(m) are elliptic integrals of the first and second order. Any coil shape can be incorporated into the model by numerically solving equation 13 [7]. Since we assume that the coil is parallel to the plane of the neurons, we can neglect the changes in the magnetic field in the z direction. Thus, we can find the induced electric field for this case (Figure 1A):
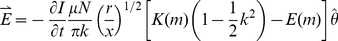
(16)


This induced electric field can be separated into a spatial function and a temporal function [11] [18]:
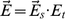
(17)where the spatial function is given by:
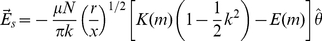
(18)and the temporal function is given by:
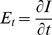
(19)


We simulated the magnetic stimulator as an RLC circuit [57]. The current in an RLC circuit can behave in two ways. In the overdamped case, the current rises to a maximum and then falls to zero. In the underdamped case, the current oscillates with decreasing amplitude [16]. For each case the current and the time derivative of the current were calculated and then used for the simulations in NEURON. For the overdamped case:
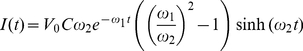
(20)


Where:
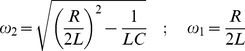
(21)where *V_o_* is the stimulus strength, i.e. the voltage that the capacitor is charged to, *C* is the capacitance, *R* is the resistance and *L* is the inductance.

Equivalently, for the underdamped case:
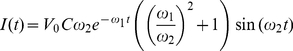
(22)


Where:
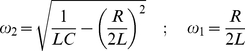
(23)


All compartmental simulations were performed with NEURON 6.2 [29], with an integration time step of 1 µs. The temporal part of the electric field (eqn. 19) was calculated in NEURON in every time step using equations 20 and 22. The spatial part of the electric field (eqn. 18) (Figure 1A) was calculated in Matlab (version 7.6.0.324 The MathWorks, Inc) prior to the simulation. This was then exported from Matlab to NEURON with a spatial resolution of 1 µm. Neuronal excitability was simulated using the Hodgkin-Huxley model [37] that is part of the NEURON simulation environment. This was also used with previously published models for neocortical pyramidal neurons [27], [38]. These models were used specifically in all simulations with realistic neuronal morphologies. Realistic morphologies were taken from [58], downloaded from www.dendrite.org, and from data from our laboratory [59]. All the conductance densities and passive membrane parameters were similar to those defined in the original models [27], [38]. Deviations from this parameter set are indicated in the relevant figure legends.
